# Structural determination of *Streptococcus pyogenes* M1 protein interactions with human immunoglobulin G using integrative structural biology

**DOI:** 10.1371/journal.pcbi.1008169

**Published:** 2021-01-07

**Authors:** Hamed Khakzad, Lotta Happonen, Yasaman Karami, Sounak Chowdhury, Gizem Ertürk Bergdahl, Michael Nilges, Guy Tran Van Nhieu, Johan Malmström, Lars Malmström

**Affiliations:** 1 Équipe Signalisation Calcique et Infections Microbiennes, École Normale Supérieure Paris-Saclay, Gif-sur-Yvette, France; 2 Institut National de la Santé et de la Recherche Gif-sur-Yvette, France; 3 Lund University, Faculty of Medicine, Department of Clinical Sciences Lund, Infection Medicine, Lund, Sweden; 4 Structural Bioinformatics Unit, Department of Structural Biology and Chemistry, C3BI, Institut Pasteur, CNRS UMR3528, Paris, France; Virginia Tech, UNITED STATES

## Abstract

*Streptococcus pyogenes* (Group A streptococcus; GAS) is an important human pathogen responsible for mild to severe, life-threatening infections. GAS expresses a wide range of virulence factors, including the M family proteins. The M proteins allow the bacteria to evade parts of the human immune defenses by triggering the formation of a dense coat of plasma proteins surrounding the bacteria, including IgGs. However, the molecular level details of the M1-IgG interaction have remained unclear. Here, we characterized the structure and dynamics of this interaction interface in human plasma on the surface of live bacteria using integrative structural biology, combining cross-linking mass spectrometry and molecular dynamics (MD) simulations. We show that the primary interaction is formed between the S-domain of M1 and the conserved IgG Fc-domain. In addition, we show evidence for a so far uncharacterized interaction between the A-domain and the IgG Fc-domain. Both these interactions mimic the protein G-IgG interface of group C and G streptococcus. These findings underline a conserved scavenging mechanism used by GAS surface proteins that block the IgG-receptor (FcγR) to inhibit phagocytic killing. We additionally show that we can capture Fab-bound IgGs in a complex background and identify XLs between the constant region of the Fab-domain and certain regions of the M1 protein engaged in the Fab-mediated binding. Our results elucidate the M1-IgG interaction network involved in inhibition of phagocytosis and reveal important M1 peptides that can be further investigated as future vaccine targets.

## Author summary

Streptococcus pyogenes is a human specific pathogen causing both mild and invasive infections. It employs sophisticated mechanisms to evade and circumvent parts of the host’s immune defenses, in part via its major surface associated virulence factor, the family of M proteins. Of these, the M1 protein is the most prevalent serotype. The M1 protein creates a dense coat-like structure with multiple host proteins on the bacterial surface to disguise itself from opsonizing antibodies. It specifically interacts in a non-immune way with human immunoglobulin G (IgG) Fc-domains to disarm their receptor binding site. The molecular level details of this interaction have not been characterized. Here, we describe these interactions from minimally perturbed samples of human plasma adsorbed onto living bacteria using an integrative structural biology approach including cross-linking mass spectrometry, molecular modeling, and molecular dynamics simulations. We identify two distinct M1-peptides that bind IgGs and reveal the stability of these interactions. We show that both peptides block the Fc-receptor binding sites through capturing IgGs via their Fc-domains. These results highlight the importance of describing novel pathogen-derived peptides mediating host immune evasion as potential vaccine targets in future studies.

## Introduction

GAS is a Gram-positive bacterium that causes both mild infections in the upper respiratory tract as well as severe, invasive systemic diseases including streptococcal toxic shock syndrome (STSS), necrotizing fasciitis (NF) and sepsis [1]. Worldwide, GAS is responsible for an estimated 700 million cases each year, of which 650,000 progress to severe invasive infections with an associated mortality of 25% [2], making GAS one of the most predominant bacterial pathogens to humans. To cause invasive infections, GAS expresses a wide range of virulence factors to evade human defense mechanisms. These virulence factors mostly consist of secreted or surface-associated proteins that target proteins and protein complexes of the innate and adaptive immune systems [3–5].

**Fig 1:**
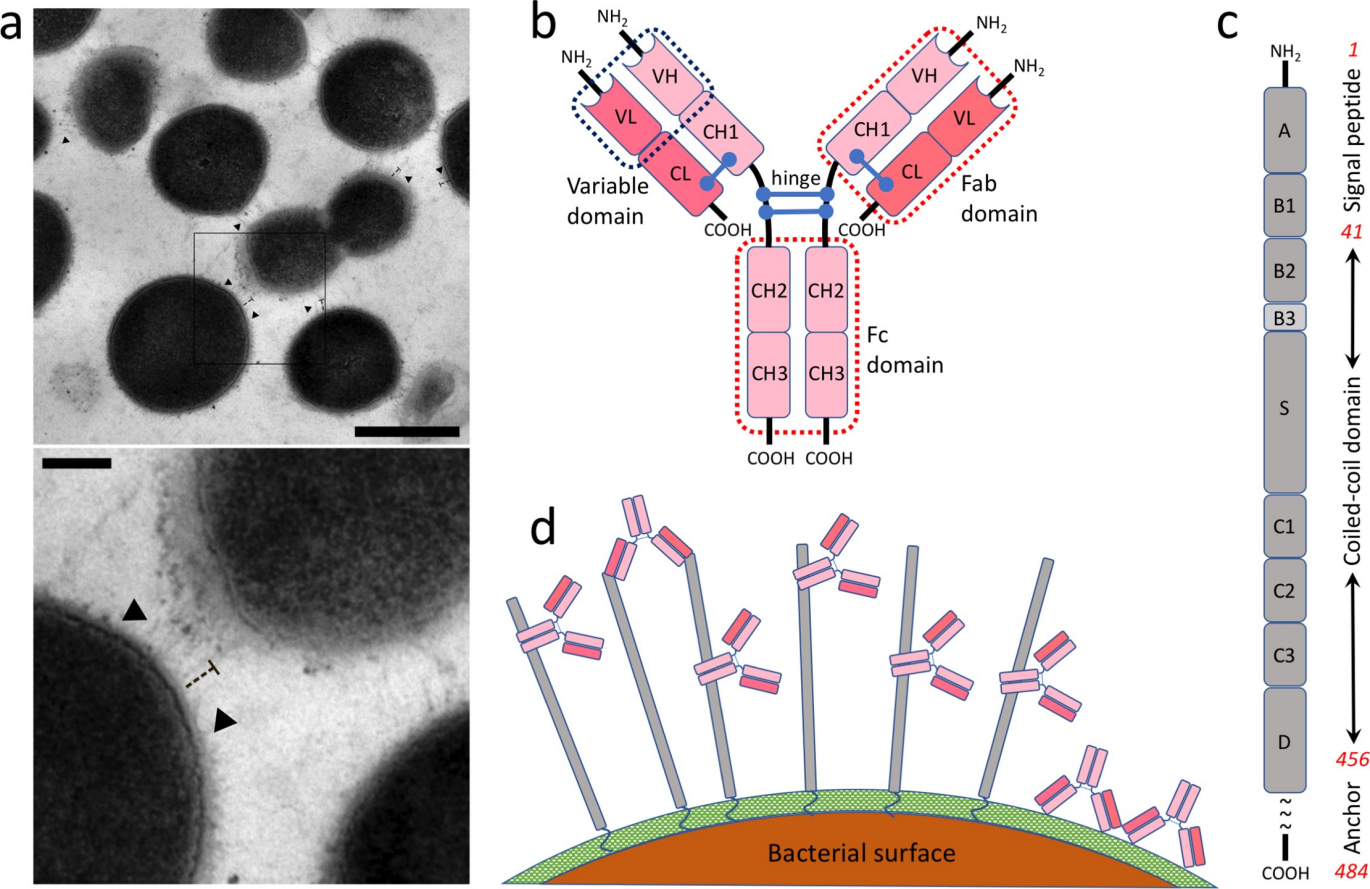
The schematic view of the studied system. **(a)** Electron microscopy image of the surface of bacteria representing the length of M1 protein. The scale bar in the larger image is 500 nm where in the zoom view below is 100 nm. Small arrows indicate the position of M1 proteins. **(b)** The general structure of human IgG. **(c)** Important domains of the M1 protein, including the hypervariable domain (A), fibrinogen binding domains (B1/B2), S-region, and albumin-binding domains (C1/C2/C3). The coiled-coil region, the anchor, and the signal peptide are shown to the right specifying by the residue numbers in red. **(d)** IgG-orientations and -interactions with the M1-protein on the surface of GAS. Three major interactions are shown, including Fc- and Fab-mediated interactions, as well as opsonizing antibodies bound to the surface of the bacteria [16]. M1 is shown in gray while IgGs are in pink. The peptidoglycan layer on the surface of bacteria is shown in green.

Specifically, GAS expresses several IgG degrading enzymes [6–8] and Fc-binding proteins [9–11] that target immunoglobulins G (IgGs), key players of the humoral immune response. In human plasma, IgG is the most abundant immunoglobulin isotype. The four subclasses IgG1-4 are highly conserved in sequence and structure, with significant non-random differences, especially in IgG3 with an extended hinge-region [12]. All subclasses are composed of two conserved heavy and light chains connected by a varying number of disulfide bonds. The heavy chain is composed of three constant domains (CH3, CH2, CH1), and one variable domain (VH) close to the N-terminus. The light chain is composed of one variable (VL) and one constant domain (CL). The CH2 and CH3 domains form the fragment crystallizable (Fc)-domain connected to CH1 via the hinge-region. Moreover, the CH1 and VH, together with the light chain VL domain, constitute the fragment antigen-binding (Fab)-domains (Fig 1).

Together with IgGs, the complement proteins of the innate immune system have a crucial role in combatting pathogens. During the classical pathway of the complement system activation, blood plasma IgGs bind to bacterial antigens via the VL and VH of the Fab-domains. The respective Fc-domains are recognized by the complement C1q, which in turn binds to Fc-receptors on phagocytic cells initializing phagocytosis. To prevent IgG-mediated opsonization and phagocytosis, GAS uses two main classes of virulence factors: the M proteins and the IgG-degrading enzymes IdeS and EndoS/EndoS2 [7,13]. In the family of M proteins, antigenic variation has resulted in more than 200 serotypes, but only a few are frequently associated with invasive disease, with M1 being the most prevalent serotype [14]. Moreover, to physically protect its vulnerable antigenic epitopes, GAS scavenges human plasma proteins to form a surrounding coat-like barrier [4,13,15], and among these interactions specifically binds IgG-molecules via their Fc-domains rendering these inaccessible for Fc-receptors [16].

Targeted cross-linking mass spectrometry (TX-MS) relies on computational structural models to score sets of targeted cross-linked peptide signals acquired using a combination of mass spectrometry acquisition techniques such as data-dependent acquisition (DDA), data-independent acquisition (DIA) and high-resolution MS1 (hrMS1). We have previously demonstrated the utility of TX-MS by creating a high-resolution quaternary model of a 1.8 MDa protein complex composed of the M1 protein and ten human plasma proteins [15]. Among these interactions, the M1-IgG binding is found to be environment-specific, binding via Fab-domains under antibody-rich conditions such as plasma, or via Fc-domains in an antibody-poor environment such as saliva [16]. We have previously shown that all human IgG subclasses can bind to the M1 protein [4]; however, the molecular details of these interactions are not fully understood.

Here, we determined the interaction of the M1 protein with human IgGs in its native environment on the bacterial surface by cross-linking the intact live bacteria in human plasma. We characterized this sophisticated mechanism in an unfractionated, complex sample using an integrative structural biology approach combining TX-MS and 10 μs MD simulations. While TX-MS helped to discover the binding sites in either protein, MD simulations revealed the accurate interaction networks and the strength of the interaction.

## Methods

### Computational modeling

The UniProt accession numbers for the M1 protein and the IgG subclasses 1–4 are Q99XV0, P01857, P01859, P01860, and P01861, respectively. Using Rosetta Software suit [17], comparative models have been generated for human IgG subclasses based on crystal structures deposited in protein data bank with PDB IDs 1hzh, 4haf, 5w38, and 4c54 for IgG1, IgG2, IgG3, and IgG4, respectively. For IgG1, we modeled the gap in the hinge region using DaReUS-Loop [18,19] while the missing residues were added using MODELLER 9.24 [20]. We then re-packed the sidechains of the full structure, and made the final set of models with rosetta relax protocol considering the disulfide bridges as the input constraints.

For computational modeling of the GAS M1 protein, we first separated the coiled-coil domain from signal peptide (N-terminal) and anchor (C-terminal), and searched them through HHpred [21] to find homologous structures (S1 Table). Then, we used the Rosetta comparative modeling protocol (RosettaCM [22]) to model each domain from its homologs separately and produced 10K models per domain. To filter out the models, we used the Rosetta energy function [17,23]. The final model is generated by another comparative modeling run, generating 10K models of the full-length protein filtered out using the Rosetta score.

### TX-MS based data analysis

To analyze the interaction of M1 and IgG1-4, we used TX-MS, which combines all three MS acquisition data (hrMS1, DDA, and DIA) with computational models. To perform the TX-MS analysis, we first developed a training set containing isotopic patterns of XLs previously identified between M1 and human plasma proteins [15]. Here, we considered 6 different features according to the isotopic patterns of the XLs; however, as we used isotope-labeled, non-cleavable DSS cross-linker in MS experiments, we could consider both heavy and light patterns for each feature. These 6 features involved monoisotopic mass, feature retention time, feature mass, intensity value, charge state, all combined with scores produced by Dinosaur, an open-source peptide feature detection software previously developed in our lab (see reference [24] for more details). We then trained an ensemble-based bagging learner using this training set and we used machine learning to identify isotopic patterns of real XLs (considering partner peptides in each XL) in hrMS1 data.

In parallel and by considering the centroid representation of each partner (side chains considered as a sphere for simplification in this stage), we used the RosettaDock protocol [25] to generate 10K flexible-backbone docking models. The models are then filtered using XLs identified by the machine learning algorithm to reveal the preliminary and potential binding interfaces between the two proteins (A- and S-domains of M1 with the CH3 domain of IgGs). The top 1% models were selected based on the number of fulfilled XLs and the average distance between all lysine residues making an XL (see below for more details).

After that, considering the lysine residues on the interface, we produced all hypothetical XLs below the cut-off threshold of 32 Å (Euclidean distance) for the selected models and evaluated them all using DDA and DIA data. For DDA analysis, we first used the monoisotopic m/z value of all generated XLs to filter out the DDA spectrums. We then produced all fragment ions per XL and searched them through the filtered DDA spectra. The same approach is applied to DIA data by converting them to DDA file format using DIA-Umpire software [26].

To score each generated model using XLs detected by either the hrMS1-based machine learning algorithm or DDA and DIA, we designed a simple weighted score based on the normal distribution of the length of the detected XLs. Considering the fact that the length of DSS is 11.4 Å and based on the literature, the maximum reliable distance to consider is ~30 Å, we considered XLs around 15–25 Å having more importance than XLs below 11.4 Å or from 25 to 32 Å. We should mention that generating the flexible-backbone docking models, as explained above, also increased and improved the flexibility of this approach here.

Finally, we re-packed the sidechains for final selected models by Rosetta docking prepack protocol and produced high-resolution docking models by considering 3 Å translation and 8° rotation. The models are then reported for each binding interface and used as input for the MD simulations.

### Cross-linking of plasma adsorption samples

For cross-linking, pooled normal human plasma was adsorbed onto the surface of *S*. *pyogenes* bacteria, as described [4,15]. The *S*. *pyogenes* M1 serotype strain SF370 from the American Type Culture Collection (ATCC; strain reference 700294), was grown at 37 °C, 5% CO_2_ to mid-exponential phase (OD_620nm_ ∼ 0.4) in TH broth supplemented with 0.3% (w/v) yeast extract. The cells were harvested by centrifugation (1900×*g*, 10 min, 22°C), washed with phosphate-buffered saline (PBS, 10 mM phosphate buffer, 2.7 mM potassium chloride, 137 mM sodium chloride (Sigma)), recentrifuged (1900×*g*, 5 min, 22°C), and resuspended to an approximate concentration of 1 × 10^9^ colony forming units  ml^−1^. Four hundred microliters of pooled normal human plasma from healthy donors (Innovative Research) supplemented with a final concentration of 10 μM argatroban (Sigma) was mixed with 100 μl of bacteria and incubated at 37 °C 30 min 500 rpm.

The bacteria with adsorbed plasma proteins were harvested by centrifugation (1900 × *g*, 5 min, 22°C) and washed twice with PBS and finally resuspended in 200 μl of PBS for cross-linking. Heavy/light disuccinimidyl-suberate cross-linker (DSS-H12/D12, Creative Molecules Inc.) resuspended in 100% dimethylformamide (Sigma) was added to final concentrations of 0.25, 0.5, 1.0 and 2.0 mM in duplicates, and incubated for 1 h, 37 °C, 900 rpm. The cross-linking reaction was quenched with a final concentration of 50 mM ammonium bicarbonate (Sigma) at 37 °C, 15 min, 900 rpm. The bacterial surface proteins with attached plasma proteins were released by limited proteolysis with 2 μG trypsin (Promega) /37 °C, 1 h, 800 rpm) prior to cell debris removal by centrifugation (1900×*g*, 15 min) and subsequent supernatant recovery. Two hundred microliters of the supernatant were recovered, and any remaining bacteria were killed by heat inactivation (85 °C, 5 min) prior to sample preparation for mass spectrometry.

### Sample preparation for mass spectrometry

The sample preparation for mass spectrometry was done as described [4,15]. Briefly, the samples were denatured in 8 M urea-100 mM ammonium bicarbonate (both Sigma), and the cysteine bonds reduced with 5 mM *tris*(2-carboxyethyl)phosphine (Sigma) (37 °C, 30 min). The cysteines were alkylated with 5 mM iodoacetamide (Sigma) (22 °C, 60 min), and the samples subsequently digested using sequencing-grade lysyl endopeptidase (37 °C, 2 h) (Wako). The samples were diluted with 100 mM ammonium bicarbonate to a final urea concentration of 1.5 M and followed by digestion using trypsin (Promega) (37 °C, 18 h). Digested samples were acidified with 10% formic acid to a pH of 3.0, and the peptides were subsequently purified with C18 reverse-phase spin columns according to the manufacturer’s instructions (Macrospin columns, Harvard Apparatus). Dried peptides were reconstituted in 2% acetonitrile and 0.2% formic acid prior to MS analyses.

### MS experiments

All peptide analyses were performed on a Q Exactive HFX mass spectrometer (Thermo Scientific) connected to an EASY-nLC 1200 ultra-high-performance liquid chromatography system (Thermo Scientific). For data-dependent analysis (DDA), peptides were separated on an EASY-Spray column (Thermo Scientific; ID 75 μm × 25 cm, column temperature 45 °C) operated at a constant pressure of 800 bar. A linear gradient from 4% to 45% of 0.1% formic acid in 80% acetonitrile was run for 50 min at a flow rate of 300 nl min^−1^. One full MS scan (resolution 60,000@200 m/z; mass range 350 to 1600 m/z) was followed by MS/MS scans (resolution 15,000@200 m/z) of the 15 most abundant ion signals. The precursor ions were isolated with 2 m/z isolation width and fragmented using higher-energy collisional-induced dissociation at a normalized collision energy of 30. Charge state screening was enabled, and precursors with an unknown charge state and singly charged ions were excluded. The dynamic exclusion window was set to 15 s and limited to 300 entries. The automatic gain control was set to 3e6 for MS and 1e5 for MS/MS with ion accumulation times of 110 and 60 ms, respectively. The intensity threshold for precursor ion selection was set to 1.7e4.

For data-independent acquisition (DIA), peptides were separated using an EASY-Spray column (Thermo Scientific; ID 75 μm × 25 cm, column temperature 45 °C) operated at a constant pressure of 800 bar. A linear gradient from 4% to 45% of 0.1% formic acid in 80% acetonitrile was run for 110 min at a flow rate of 300 nl min^−1^. A full MS scan (resolution 60,000@200 m/z; mass range from 390 to 1210m/z) was followed by 32 MS/MS full fragmentation scans (resolution 35,000@200 m/z) using an isolation window of 26 m/z (including 0.5 m/z overlap between the previous and next window). The precursor ions within each isolation window were fragmented using higher-energy collisional-induced dissociation at a normalized collision energy of 30. The automatic gain control was set to 3e6 for MS and 1e6 for MS/MS with ion accumulation times of 100 ms and 120 ms, respectively.

For high-resolution MS1 (hrMS1), peptides were separated using an EASY-Spray column (Thermo Scientific; ID 75 μm × 25 cm, column temperature 45 °C) operated at a constant pressure of 800 bar. A linear gradient from 4% to 45% of 0.1% formic acid in 80% acetonitrile was run for 60 min at a flow rate of 300 nl min^−1^. High-resolution MS scans (resolution 240,000@200 m/z; mass range from 400 to 2000m/z) were acquired using automatic gain control set to 3e6 and a fill time of 500 ms.

### Biacore experiment

Binding experiments were carried out using Biacore X100 (Cytiva Life Sciences, Uppsala, Sweden) with control software version of v.2.0. Sensor chip CM5 (Cytiva Life Sciences, Uppsala, Sweden) was used as gold chips and all the assays were carried out at 25°C. Amine coupling kit (Cytiva Life Sciences, Uppsala, Sweden) which was containing EDC [1-Ethyl-3-(3-dimethylamino-propyl)carbodiimide] (75 mg/mL), NHS (N-hydroxysuccinimide) (11.5 mg/mL) and ethanolamine (1 M, pH: 8.5) was used for the covalent immobilization of M1 via amine groups on the gold surface. Before starting the immobilization procedure, CM5 chip was docked into the instrument and the chip surface was activated following EDC/NHS protocol with PBS as the running buffer. The ligand (M1) at a concentration of 0.01 mg/mL (in 10 mM acetate buffer, pH: 5.0) was injected for 7 min (flow rate: 10 μL/min) followed by a 7 min (flow rate: 10 μL/min) injection of 1.0 M ethanolamine in order to deactivate excess reactive groups. Immobilization procedure was completed after the targeted immobilization level (≈ 2500 RU) was reached.

Only the flow channel_2 (active channel) was used for the ligand immobilization while the flow channel_1 (reference channel) was used as a reference to investigate non-specific binding. Subtracted channel (flow channel_2—flow channel_1) was used to evaluate the results of the analysis. Commercial IgG1—Xolair (Omalizumab, 150 mg, Product code 07612797314975, Lot Number AVXS239902) was injected into the active and reference channels at concentrations of 0, 0.009375, 0.01875, 0.0375, 0.075, 0.15 and 0.3 μM, respectively. Triplicate injections were done for each concentration. Association time was set to 120 s while the dissociation time was kept for 600 s. Flow rate was 10 μL/min and 10 mM glycine-HCl (pH: 2.5) was used as the regeneration buffer. Response units recorded from the subtracted channel (flow channel_2—flow channel_1) was used to evaluate the results of the analysis.

### Evaluation of the Biacore data

For the evaluation, the parameters were determined by Biacore Evaluation Software v.2.0 in binding analysis and kinetic evaluation based on curve-fitting algorithms which employs global fitting. Collected data for the experiment was analyzed in one fit using the kinetic fitting programs that yields ka, kd and KD values. Equilibrium binding analysis was performed by plotting the RU values measured in the plateau for each concentration and fitting the data to one of the binding models. First the binding was tested for the simplest 1–1 Langmuir binding model which follows the equation:

$$A+B\begin{array}{c} ka\\ ↔\\ kd \end{array}AB$$

where A is the analyte, B is the ligand, AB is the complex. The ka (rate of association, M^-1^s^-1^) is measured from the reaction in the forward direction while the kd (dissociation rate, s^-1^) is measured from the reverse reaction. The binding was also tested for heterogeneous ligand model where the same analyte binds independently to multiple ligands or to several binding sites on the same ligand. Heterogeneous ligand model follows the equations:

$$A+B1\begin{array}{c} {ka}_{1}\\ ⇌\\ {kd}_{1} \end{array}AB1$$

$$A+B2\begin{array}{c} {ka}_{2}\\ ⇌\\ {kd}_{2} \end{array}AB2$$

where A represents the analyte, B1 and B2 represent two different ligands or two different binding sites on the same ligand, respectively, AB1 and AB2 represent the first and second complexes formed after the binding of the analyte to the surface, ka_1_ and ka_2_ are the association rates of the first and second complexes while kd_1_ and kd_2_ represent the dissociation rates.

### MD simulations

We performed MD simulations on the two complexes of M1(S-domain peptide)-IgG1(Fc) and M1(A-domain peptide)-IgG1(Fc). The starting conformations correspond to two different binding sites for the Fc domain of IgG1 (residues T108-K330) on the protein M1: (*i*) residues L108-K123 of M1(A-domain) with peptide sequence: LETKLKELQQDYDLAK, and (*ii*) residues G208-Q223 of M1(S-domain) with peptide sequence: GNAKLELDQLSSEKEQ. MD simulations were carried out with the Gromacs 2018.3 [27] using the amber99sb forcefield parameter set: (*i*) Na^+^ and Cl^-^ counter-ions were added to reproduce physiological salt concentration (150 mM solution of sodium chloride), (*ii*) the solute was hydrated with a triclinic box of explicit TIP3P water molecules with a buffering distance of up to 12 Å, and (*iii*) the environment of the histidine was checked and they were consequently protonated with a hydrogen at the ε nitrogen.

First, we performed 5000 steps of minimization using the steepest descent method keeping only protein backbone atoms fixed to allow protein side chains to relax. After that, the system was equilibrated for 300 ps at constant volume (NVT) and for further 1 ns using a Langevin piston (NPT) [28] to maintain the pressure, while the restraints were gradually released. For every complex, five replicates of 1 μs, with different initial velocities, were performed in the NPT ensemble. The temperature was kept at 310 K and pressure at 1 bar using the Parrinello-Rahman barostat with an integration time step of 2.0 fs. The Particle Mesh Ewald method (PME) [29] was employed to treat long-range electrostatics, and the coordinates of the system were written every 100 ps. The root mean square deviations (RMSD) of the studied complexes from the equilibrated structure were measured on the backbone (C, Ca, N, O) atoms along simulation time for all the replicates (S4 Fig and S2 Table). All systems were fully relaxed after 100 ns. Consequently, the last 900 ns of each replicate were retained for subsequent analyses. Moreover, we calculated the residue root mean square fluctuations (RMSF) over the last 900 ns of every simulation (S5 Fig and S2 Table) with respect to the average conformation and over the backbone (C, Ca, N, O) atoms.

### COMMA2 analysis

For every studied system, COMMA2 was applied to the last 900 ns of the five replicates of MD simulations, and communication blocks were extracted. COMMA2 identifies pathway-based communication blocks (CBs^path^), *i*.*e*., groups of residues that move together, and are linked by non-covalent interactions, and clique-based communication blocks (CBs^clique^), *i*.*e*., groups of residues that display high concerted atomic fluctuations, and that are close in 3D space (see [30,31] for formal definitions and detailed descriptions).

### Thin section electron microscopy

Pelleted SF370 cells were resuspended in EM fixative (1.5% glutaraldehyde and 1.5% paraformaldehyde in 0.1 m Sörensen's phosphate buffer pH 7.2) and incubated at room temperature for 1h. After fixation, the cells were rinsed using 0.1 m Sörensen's phosphate buffer pH 7.2, postfixed in 1% osmium tetraoxide (OsO4) for 1h, dehydrated using increasing concentrations of acetone and finally embedded in Polarbed 812 (Polaron). Ultrathin sections (50–60 nm) were prepared using a Leica EM UC7 ultramicrotome and placed on thin-bar copper grids (Maxtaform H5). The sections were stained with 4% uranyl acetate at 40°C and 0.5% lead citrate at room temperature. The prepared thin sections were examined with a Technai G2 Spirit electron microscope (FEI, Eindhoven, The Netherlands), operating at an excitation voltage of 100kV, and equipped with a Veleta (Olympus) 2kx2k CCD-camera. The length measurements of the surface attached M1 proteins were done using the Veleta software.

## Results

### TX-MS structural analysis

Here, we elucidated the complex network of M1-IgG interactions *ex vivo* arising in pooled normal human plasma adsorbed on the surface of live bacteria, mimicking a scenario during invasive infections. Fig 1 shows a schematic representation of the applied approach. To study the interaction of dimeric, coiled-coil multidomain (domains A-D, Fig 1C) M1 protein with human IgGs, we incubated live bacteria in human plasma, allowing for Fab- and Fc-mediated binding of the plasma IgG-molecules to the M1 protein or the opsonic, Fab-mediated binding to the surface of the bacteria (Fig 1D). A mutant strain (ΔM1) lacking the M1 protein was used as a negative control (S1 Fig). The formed, native interactions were captured by chemical cross-linking followed by mass spectrometric identification of cross-linked peptides (see Methods). To map the identified XLs into the structure and provide molecular details of the interaction, we produced comparative models for the M1 protein and the full-length IgG1, using RosettaCM protocol, as explained in the Methods section. The length of the coiled-coil domain in the final model provided for the M1 protein was 489.5 Å matching with our observation based on electron microscopy images represented in Fig 1A, where the average length of the M1 protein on the SF370 surface as measured from the cell wall was 48 ± 7 nm (n = 15) in epon embedded samples. With that said, to our knowledge, this is the first accurate full-length model of M1 protein in terms of the length and the shape of the coiled-coil domain.

An initial DIA analysis of pooled human plasma proteins absorbed to the streptococcal surface revealed that the four subclasses of IgG are highly abundant with different intensities in the MS samples (S1 Fig). The measured abundance distribution deviates from the distribution in human plasma (IgG1 > IgG2 > IgG3 > IgG4) [12]. These results confirmed that IgG1 and IgG3 are the most abundant IgGs bound to S. pyogenes and that IgG3 is enriched on the surface of the wt strain compared to the deltaM1 mutant, due to a combination of Fab- and Fc-bound IgGs. Moreover, data analysis of DDA samples using the sequence of the M1 protein and the heavy chains of all IgG subclasses resulted in the identification of several inter- and intra-cross-links (XLs). Accordingly, we obtained 21 distinct XLs, of which 17 are inter XLs (between the M1 protein and the different IgG subclasses), which were considered for modeling refinement. The majority of inter XLs (10 out of 17) supported the Fc-mediated binding of IgGs to M1, while the rest indicated novel Fab-mediated interactions supported by XLs between the M1 protein and the constant region of the Fab-domain. The presence of specific M1 IgG-molecules in commercial pooled normal human plasma has been described [4]; however, the specific epitopes they target has remained unknown.

**Fig 2:**
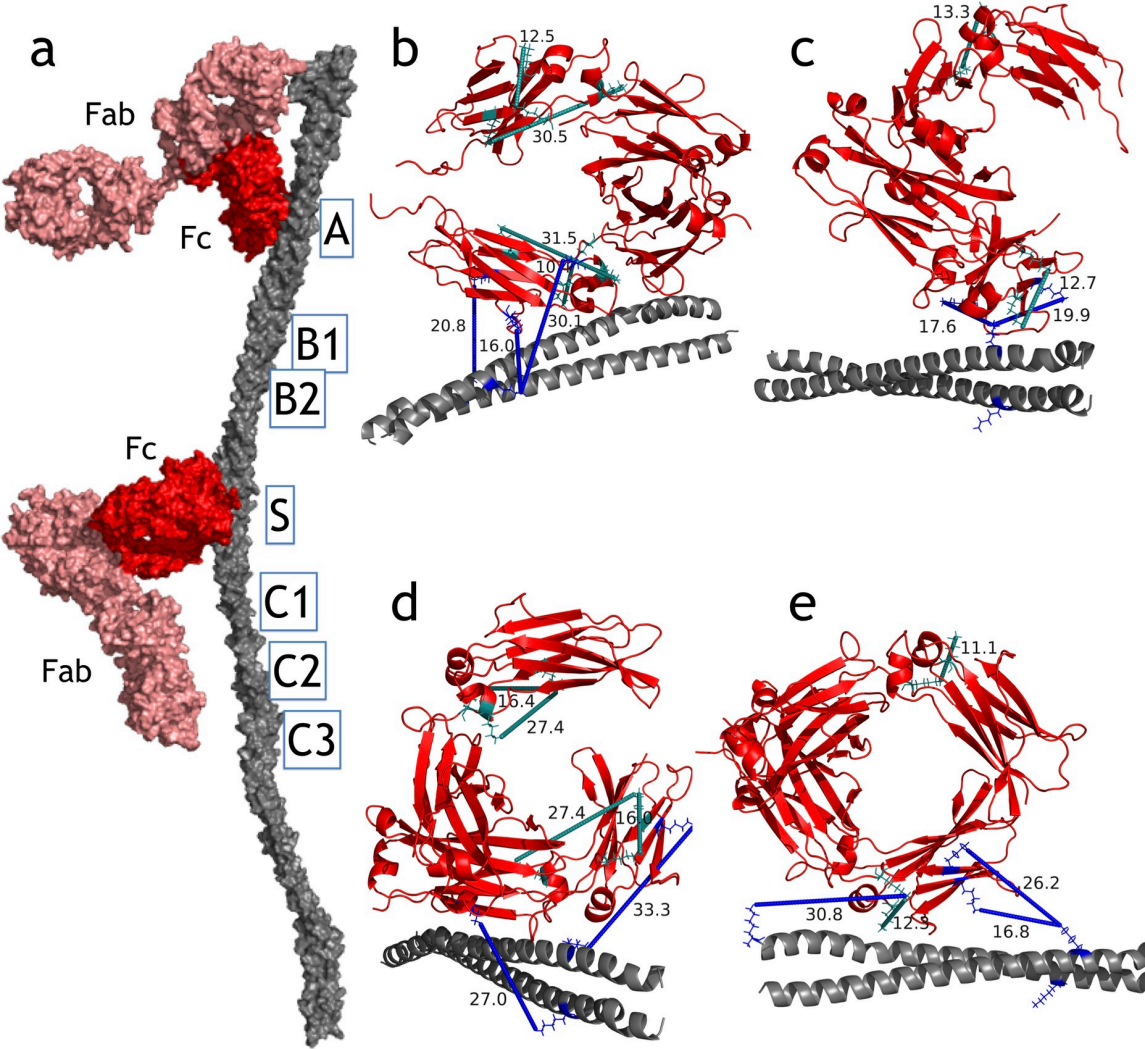
M1 interactions with IgG subclasses. **(a)** The model for M1 (A- and S-domains)-IgG1 full length non-immune Fc-mediated interaction. Important domains of the M1 protein are shown, where A- and S-domains play a crucial role. The molecular details of the Fc-binding site are shown in panel **(b)** where inter- and intra-XLs are mapped on the protein complex. **(b-e)** The S-domain of the M1-protein interacts with the Fc-region of IgG1-4. All models are generated by the TX-MS workflow based on cross-link constraints derived from MS data. Intra-XLs for each IgG are shown in cyan, while inter-XLs between the two proteins are in blue. The binding interfaces of the M1 protein on the Fc-domain of all IgG subclasses are similar and mediated via the CH3 domains. This region is involved in binding to IgG Fc-receptors (FcγR), predicted to be inhibited by the M1 protein interaction.

**Fig 3:**
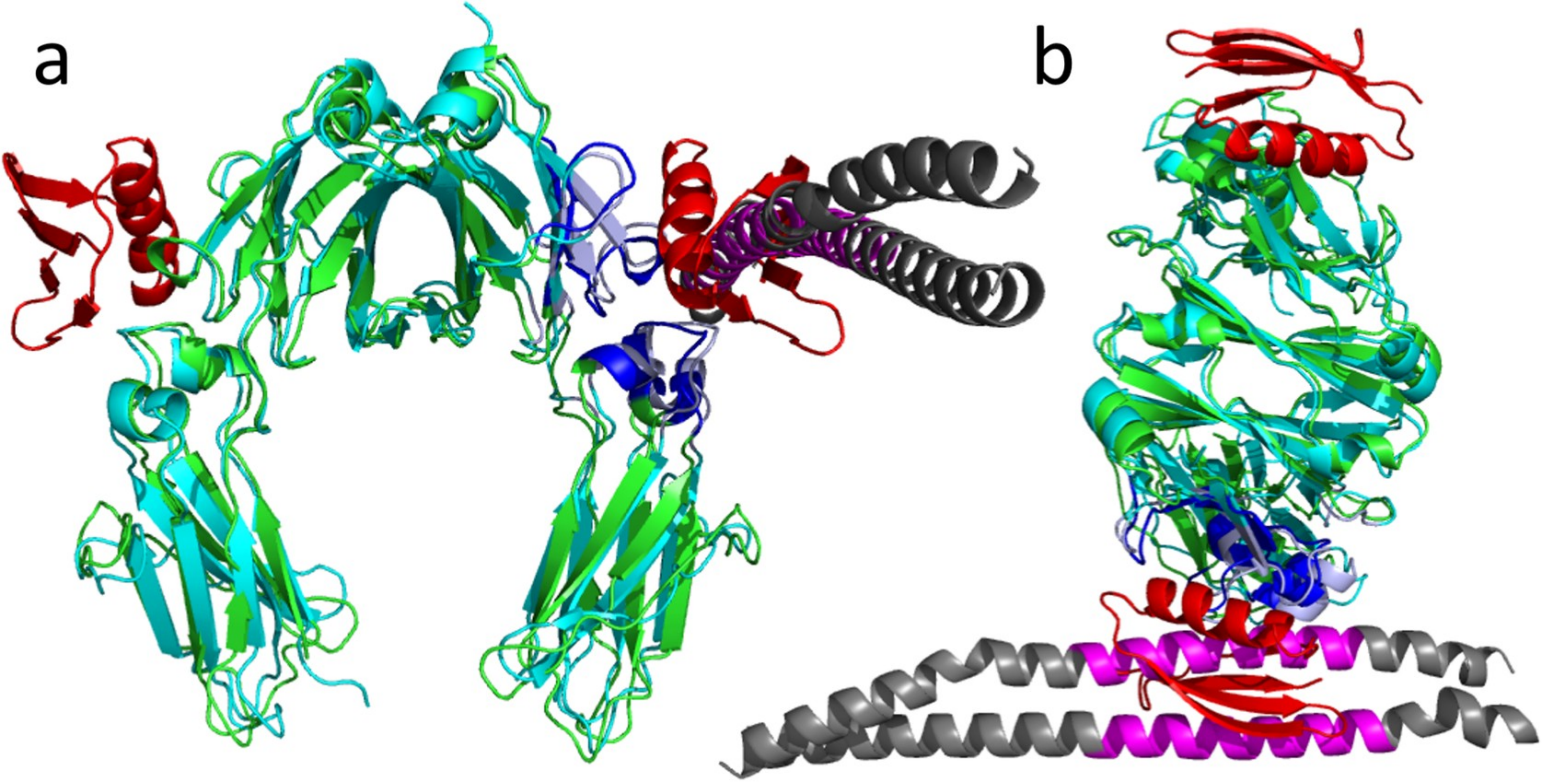
The TX-MS based M1-IgG model aligned with that of the streptococcal protein G. **(a-b)** Protein G (PDB ID: 1FCC) is shown in red with its binding interface on IgG1 in dark blue. The M1 protein is shown in magenta and dark grey with its binding interface on IgG in light blue. Both proteins share the same binding site on IgGs in CH3 domains and close to CH2.

To elucidate the Fc-binding interface, we used TX-MS based on hrMS1 and DDA data that we combined with previously determined crystal structures of human IgGs and the partial peptides of M1 (A- and S-domains) [15] (see Methods). As demonstrated in Fig 2, all IgG subclasses bound to the M1 protein through a specific site in the IgG CH3 domain, which is essential for binding to FcγR receptors. This IgG-binding site has previously been shown to bind to a variety of proteins other than Fc-receptors, including the streptococcal protein G [32,33] and the staphylococcal protein A [34], both of which are commercially available as tools to capture IgG-molecules via their Fc-domains. Fig 3 shows the binding-site comparison between the M1-IgG1 model and the C2 fragment of the streptococcal protein G on the IgG Fc-domain. Accordingly, both M1 and protein G share the same binding site on the IgG Fc-domain and bind to all IgG subclasses [33].

**Fig 4:**
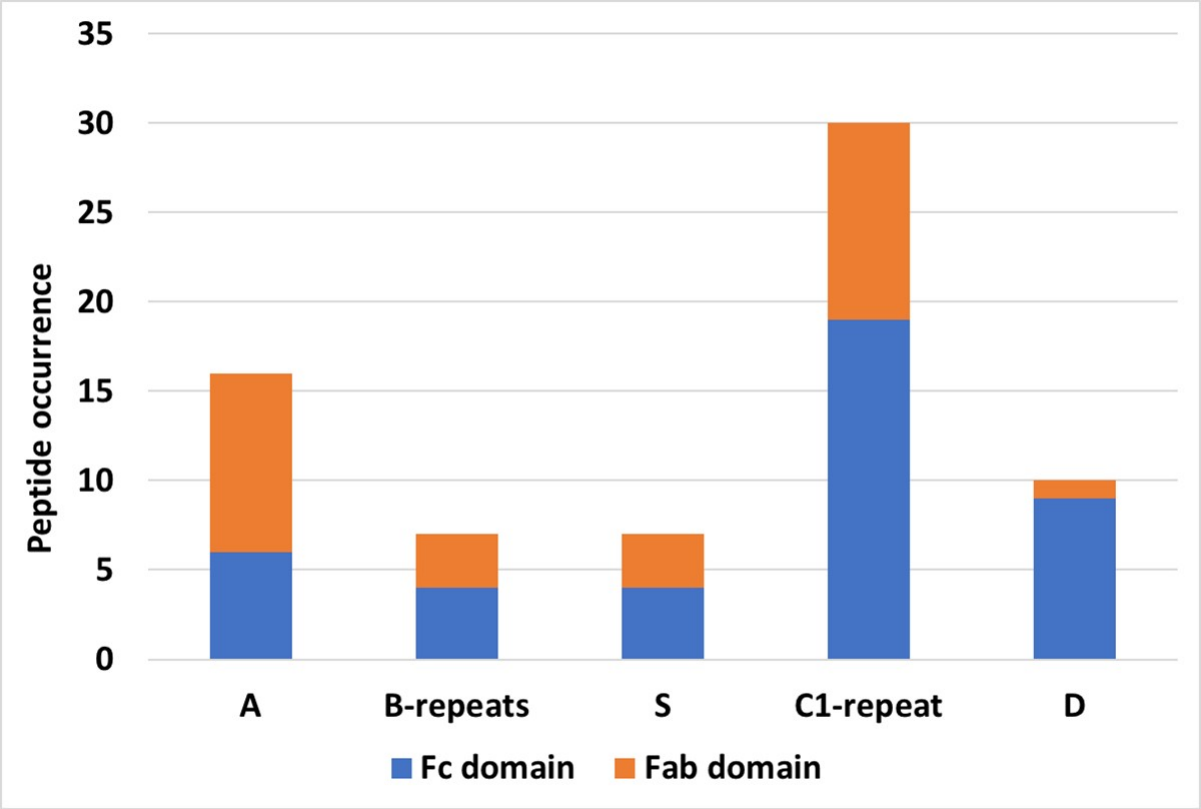
DDA-based peptide quantification of Fc and Fab fragments of IgG subclasses bind to different sub-domains of M1 protein. All IgG subclasses bind to the M1 protein through their Fc-domains. The S- & C1-domains and the hypervariable domain A of the M1 protein have the highest affinity for IgG subclasses. Only IgG heavy chains were considered.

We have additionally made a high-resolution model for the full-length IgG1 interaction with the M1 protein. The model, shown in Fig 2, interestingly supports Fab-mediated binding as well, either facilitated by the Fc-binding positioning the Fab-domain in close proximity to the M1 protein leading to the formation of an XL, or by specific, Fab-mediated immune binding. The latter binding mode is supported by our previous findings, according to which pooled normal human plasma contains M protein-specific IgG antibodies [4]. Here, we hence extend our earlier results to include the identification of the most common regions of the M1 proteins that are engaged in Fab-mediated binding. In addition to the XLs supporting Fc-mediated binding, which is mostly converged to the A- and C1-domains of M1, the immune Fab-interaction is detected on the A- and C1-domains of the M1 protein based on the TX-MS data (Fig 4).

### Comparison of IgG subclasses by XL-MS quantification

Analyzing the DDA data revealed 17 inter-XLs between the M1 protein and the different subclasses of IgGs. However, the distribution of XL-peptides differed between samples with different DSS concentrations. While some of the XLs were detected in all samples (e.g., XLs in Fig 2), others were less frequent. We took advantage of this, and quantified the number of detected XL peptides per acquisition specific to each IgG subclass, disregarding that some peptides are identical between all subclasses (S2 Fig). Based on this XL-peptide quantification, two regions of the M1 protein were identified with the highest number of detectable M1-IgG XLs: i) the hypervariable domain A and ii) the S- & C1-domains (end of the S-domain and beginning of the C1-repeat). These important domains of the M1 protein are highly conserved in most types of M proteins [35]. While most of the peptides from the Fc-domain were linked to the S- and C1-domains, VH located Fab-domain peptides were linked to the A-domain as well. Fig 4 shows the quantification results, where the importance of the C1-repeat and A domain is evident. **Table 1** contains a list of important peptides in the binding interfaces sorted by their occurrences in cross-linking data.

**Table 1:** Peptides with high-affinity binding occurrences. The listed peptides are frequent in the binding interfaces of the M1 protein and different IgGs according to the XL-MS quantification.

Peptide	Protein	Domain	peptide Occurrence
EEKQISDASR	M1	C1-region	12
LKELQQDYDLAK	M1	A	4
ANVLEKELETITR	M1	B2-B3-S	3
ATALEKELEEK	M1	B1-B2 repeats	2
TKGQPR	IgG2, IgG3	Fc	6
ALPAPIEKTISK	IgG1, IgG3	Fc	5
AKGQPR	IgG1, IgG4	Fc	4
CKVSNK	IgG1-4	Fc	2
ASTKGPSVFPLAPCSR	IgG2, IgG3, IgG4	Fab	5
VDKTVER	IgG2	Fab	4
PSNTKVDK	IgG1-4	Fab	4

Interestingly, the detected peptide from the A-domain is homologous to the streptococcal protein G helix, shown to bind IgG1 (Fig 3 and S3 Fig). The B-repeats had the fewest number of XLs; however, we identified XLs between B1- and B2-repeats and human plasma fibrinogen in all samples. Fibrinogen is an abundant plasma protein known to bind to the B-repeats of the M proteins [14,15]. Our results suggest that fibrinogen can mask the IgG epitopes in this region or that very few IgG binds to this region, as indicated in previously published work [36].

### Communication networks

**Fig 5:**
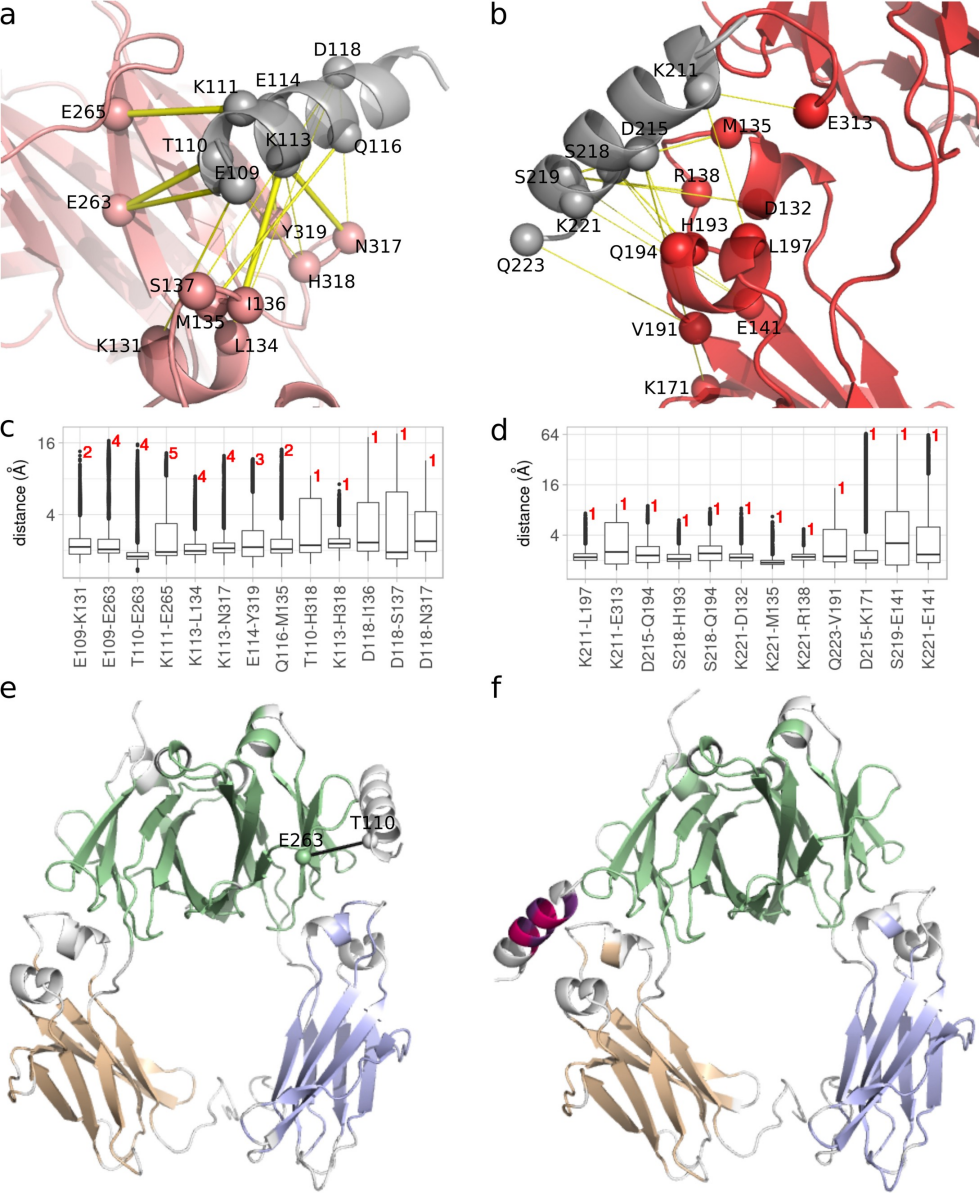
H-bond network between the M1 peptides and IgG Fc-domain. The residues forming H-bond contacts between the **(a)** M1(A)-IgG(Fc) and **(b)** M1(S)-IgG(Fc) are shown with spheres. The thickness of the yellow lines corresponds to the persistence of the interactions throughout the replicates of MD simulations. The M1 peptide is colored in gray while the chains of the IgG Fc-domain are depicted in red and pink. The distribution of distances between the residues forming H-bond contacts along the MD simulations are shown for the (**c**) M1(A)-IgG(Fc) and (**d**) M1(S)-IgG(Fc). The number of replicates in which, the H-bonds are formed for at least 40% of the simulation time are added in red at the top of each box. Communication Blocks (CBs^path^) identified by COMMA2 are mapped on the structure of **(e)** M1(A)-IgG(Fc), and **(f)** M1(S)-IgG(Fc). Distinct CBs^path^ of four residues or more are highlighted in different colors. The isolated communication pathway between the M1 peptide A-domain (T110) and IgG Fc-domain (E263) is depicted by a black line on the corresponding structure.

To further evaluate the affinity, and stability of the binding interface and to understand the interaction networks between the M1 protein and IgG molecules, we first performed Surface plasmon resonance (SPR) measurements using recombinant M1 protein and a commercial monoclonal IgG1 that only bind via Fc. These measurements showed that the Fc binding is associated with a heterogeneous ligand model [37] with two binding sites between the M1 protein and IgG-Fc with an affinity of 3,19x10^-8^ M and 4,31x10^-8^ M respectively (see S3 Table, S1 Text and S6 Fig for more details). In the following step we performed five replicates of 1 μs MD simulations starting from each of the two models for M1(S-domain peptide)-IgG1(Fc) and M1(A-domain peptide)-IgG1(Fc), resulting in a total of 10 μs MD simulations. Based on the simulations, the M1(S)-peptide was stable in three replicates (S1, S3 and S4 Movies), while in the other two replicates, it completely detached from the Fc-domain after about 100–150 ns (S2 and S5 Movies). On the other hand, the M1(A)-peptide remained stable with the Fc-domain along the simulation time of all the five replicates (S6–S10 Movies). Next, we performed hydrogen bond (H-bond) analysis over the replicates of each system and recorded those that are present at the interface between the IgG1(Fc)- and M1-peptides for at least 40% of the simulation time (S4 Table). This analysis revealed a strong network of interactions between the M1(A)-peptide-IgG1(Fc), supported by several H-bonds that are persistent among the five replicates. However, we obtained a less stable or transient binding between the M1(S)-peptide-IgG1(Fc) supported by H-bonds that are present only in one replicate. In total, we recorded 33 H-bonds for M1(A)-peptide-IgG1(Fc), with respect to 12 H-bonds for M1(S)-peptide-IgG1(Fc). These interactions are mapped on the structure of both complexes as yellow edges (Fig 5A and 5B), where the thickness of the edges correspond to the intensity and persistence of the H-bonds among the replicates. Moreover, we recorded the distances between the pairs of residues forming those H-bonds from the corresponding replicates along the simulation time (Fig 5C and 5D). The results support the strong interactions between the M1 A-domain peptide and IgG Fc-domain.

Moreover, for each system, we applied COMMA2 to extract communication blocks, *i*.*e*., CBs^path^ (see Methods). The residues comprised in a CB^path^ are linked by communication pathways by transitivity. A communication pathway is defined as a chain of residues displaying correlated motions and linked by stable non-covalent interactions. Hence, it represents an efficient route of information transmission supported by physical interactions. COMMA2 analysis revealed three different CBs^path^ for each system (Fig 5E and 5F), of which one (in green) contains both CH3 domains of Fc-domain. Two other CBs^path^ are formed (in purple and sand color), one over each CH2 domain. The results suggest a similar and pertinent pattern for the IgG Fc-domain. However, differences for the M1 peptides can be noticed. Interestingly, as shown in (Fig 5E), one residue from the M1(A)-peptide (T110), forms a short communication pathway with another residue from the Fc-domain (E263). This pathway fits the definition of isolated communication from a previous study [31], supporting a critical communication between the M1(A) peptide and IgG1(Fc). On the other hand, two small CBs^path^ are detected on the M1 S-domain peptide, as depicted in Fig 5F, that could be explained by the independent dynamics of the S-domain peptide with respect to the Fc-domain. Since the peptide does not interact with the Fc-domain, it remains more stable and can form intra communications.

## Discussion

In this study, we characterized the GAS-M1 human-IgG interactions using TX-MS combined with MD simulations. Accordingly, TX-MS revealed the two binding interfaces, while the MD simulations helped to elucidate the interaction network in molecular detail. We identified that the M1 protein is capable of capturing human IgGs to prevent opsonization. All IgG subclasses bound to the M1 protein in a specific region in the CH3 domain and close to CH2, which is involved in binding IgG-receptors (FcγR). This CH3 binding site has previously been shown to bind streptococcal protein G [32,33]. Here, the interaction network revealed by MD simulations indicated that both the M1 protein and the streptococcal protein G share the same binding site on the IgG molecules. This interaction would mask the recognition site for FcγR receptors, hence protecting the bacteria from phagocytic killing.

The structural details of the M1 protein in complex with human IgGs revealed important peptides at the binding interface that plays a crucial role in the interactions. We quantified the number of detected XL peptides per sample for each IgG subclass and identified two domains of the M1 protein associated with the highest number of XLs, namely the hypervariable A- and the S-domains (together with the beginning of the C1-repeat). Moreover, our 10 μs MD simulations put in evidence a transient binding for the M1(S)-peptide, and a stable binding for the M1(A)-peptide to the IgG1(Fc)-domain. These results indicated that the two specific peptides of the M1 protein could effectively inhibit the binding of FcγR receptors, making them potential vaccine candidates for future studies.

Most vaccine developments and trials targeted for *S*. *pyogenes* are currently focused on the M proteins and their fragments, with the leading ones targeting the N-terminal or the C-repeat region [38]. We have previously shown that peptides in the M protein C-region containing the EEKQISDASR-motif (**Table 1**) are potent epitopes for opsonizing antibodies and crucial for the interaction and internalization with monocytic cells; a result corroborated here [4]. Our data also indicates that epitopes in the A-domain are central in both Fab- and Fc- IgG1-mediated evasion of the human immune responses, and potential novel targets for therapeutic strategies to combat GAS infections.

## Conclusions

GAS is an important human pathogen infecting more than 700 million individuals globally each year. Here, we describe the interaction of the M1 protein, an important virulence factor of GAS, with human immunoglobulin G (IgG1-4) using targeted cross-linking mass spectrometry combined with molecular dynamic simulations. These interactions revealed that all IgG subclasses could bind to the M1 protein with their non-immune Fc-domains and share roughly the same Fc-binding interface on the Fc-receptor-binding domain, showing the crucial role of the M1 protein to eliminate IgG-Fc receptors (FcγR) interactions and protect the bacteria from phagocytic killing. Finally and as the result of this study, the highly frequent peptides in the important C1-repeat (EEKQISDASR), the transient binding peptide of S-domain (GNAKLELDQLSSEKEQ), and the strong binding peptide in the hypervariable A-domain (LETKLKELQQDYDLAK) on the interface of the M1 protein and IgGs can be used as vaccine candidates for further studies.

## Supporting information

S1 Text Biacore analysis of IgG1 binding to immobilized M1. (DOCX)

S1 Table Identified homologous structures for the M1 protein domains. The list of homologous proteins identified by HHpred for each domain of the M1 protein that are used in the comparative modeling of the full-length M1.
(XLSX)

S2 Table RMSD and RMSF values for the studied system in different replicates. The average and standard deviations of the RMSD and RMSF values are recorded along the MD simulations for every chain of each replicate.
(XLSX)

S3 Table Kinetic constants that are calculated via the heterogeneous ligand model. ka_1_: association rate constant of the first complex, ka_2_: association rate constant of the second complex, kd_1_: dissociation rate constant of the first complex, kd_2_: dissociation rate constant of the second complex, KD_1_: affinity constant of the first complex, KD_2_: affinity constant of the second complex (ka: M^-1^s^-1^, kd: s^-1^, KD: M). Kinetic constants were reported as the mean value of triplicate analysis.
(XLSX)

S4 Table H-bonds formed between the M1 peptides and IgG1 Fc-domain. The persistence of the H-bonds formed at the interface are recorded as a percentage over the MD simulation time. For each studied system, the average values and the number of replicates over which the H-bonds were present, are reported.
(XLSX)

S1 Fig TIC normalized intensity of human IgG subclasses in plasma samples. The intensity level of the heavy chains of human IgGs is analyzed through a DIA-MS analysis approach. Two groups of samples are considered here: pooled normal human plasma on the surface of the wt strain SF370 and on the surface of an SF370-derived M1 mutant strain (deltaM1). The heavy chains of IgG3 and IgG4 (IGHG3 and IGHG4, respectively) have the highest and the lowest intensities among all IgG subclasses. The data also indicates that S. pyogenes has a high affinity for IgG1 and IgG3, which is M1-mediated.
(TIF)

S2 Fig Multiple sequence alignment of all IgG subclasses. Identified XLs are shown with red dashed boxes where small sequence differences can be seen. The main difference can be noticed between IgG3 and other subclasses as the longer hinge region of IgG3 (residues 100–150 in IgG3).
(TIF)

S3 Fig Sequence similarity between protein G helix and the detected peptide of M1-A domain. **(a)** Crystal structure of protein G and human IgG1 (PDB id 1fcc). **(b-c)** Structural and sequence alignment of protein G helix (in red) on the peptide from the M1-A domain (in grey) detected by cross-linking mass spectrometry as the high-affinity peptide to bind IgGs.
(TIF)

S4 Fig The root mean square deviations for M1(S)-IgG(Fc) and M1(A)-IgG(Fc). The RMSD from the equilibrated structure is computed on the backbone (C, Ca, N, O) atoms and averaged over all the five replicates of **(a)** the M1 peptides and (**b** and **c**) the two chains of Fc. The average values are shown as lines and the shades correspond to the standard deviations, with blue for M1(A)-IgG(Fc) and red for M1(S)-IgG(Fc).
(TIF)

S5 Fig The root mean square fluctuations for M1(S)-IgG(Fc) and M1(A)-IgG(Fc). The RMSF was measured on the backbone (C, Ca, N, O) atoms with respect to the average conformation and averaged by residue, considering the last 900 ns of the MD simulations for **(a)** the M1 peptides and **(b**-**c)** the two symmetrical chains of Fc. The values are averaged over the five replicates of M1(A)-IgG(Fc) in blue and M1(S)-IgG(Fc) in red. The average values are shown as lines and the shades correspond to the standard deviations.
(TIF)

S6 Fig The Biacore analysis of IgG1 binding to immobilized M1. **(a)** Sensorgrams representing the response unit (Y-axis) plotted as a function of time (X-axis) for IgG1 binding to immobilized M1. **(b)** Calibration curve that shows the response unit (RU)(Y-axis) vs. IgG1 concentration (X-axis). **(c-d)** Kinetic analysis of IgG1 binding to immobilized M1 fitted to different models. **(c)** IgG1 binding fitted to 1–1 model, and **(d)** IgG1 binding fitted to heterogeneous ligand model.
(TIF)

S1 Movie 1 μs MD simulation of M1(S)-IgG1(Fc), first replicate. (MP4)

S2 Movie 1 μs MD simulation of M1(S)-IgG1(Fc), second replicate. (MP4)

S3 Movie 1 μs MD simulation of M1(S)-IgG1(Fc), third replicate. (MP4)

S4 Movie 1 μs MD simulation of M1(S)-IgG1(Fc), fourth replicate. (MP4)

S5 Movie 1 μs MD simulation of M1(S)-IgG1(Fc), fifth replicate. (MP4)

S6 Movie 1 μs MD simulation of M1(A)-IgG1(Fc), first replicate. (MP4)

S7 Movie 1 μs MD simulation of M1(A)-IgG1(Fc), second replicate. (MP4)

S8 Movie 1 μs MD simulation of M1(A)-IgG1(Fc), third replicate. (MP4)

S9 Movie 1 μs MD simulation of M1(A)-IgG1(Fc), fourth replicate. (MP4)

S10 Movie 1 μs MD simulation of M1(A)-IgG1(Fc), fifth replicate. (MP4)
