# Supplementary material for: Structural determination of Streptococcus pyogenes M1 protein interactions with human immunoglobulin G using integrative structural biology
Source: PLoS Comput Biol. 2021 Jan 7;17(1):e1008169. doi: 10.1371/journal.pcbi.1008169 (PMC7817036; doi:10.1371/journal.pcbi.1008169)
Supplement: S1 Text — (DOCX) [file pcbi.1008169.s001.docx]

**Biacore analysis of IgG1 binding to immobilized M1**

The binding of IgG1 to M1 as depicted in the sensorgrams (S6a Fig), shows that IgG1 is directly binding to M1 in a concentration dependent manner with a good correlation and good fitting to logarithmic regression (S6b Fig, R^2^= 0.96). For the kinetic analysis, the data was first tested for fitting to the simplest Langmuir 1-1 binding model (S6c Fig). However, a better fit was obtained when the data was tested for heterogeneous ligand model (S6d Fig). Using the heterogeneous ligand model the recorded chi^2^ (curve fidelity) was lower than the chi^2^ recorded for 1-1 model and the residuals which correspond to the difference between the actual and fitted data, were within ± 5% of Rmax, indicating a better fit. The fitted lines represent a better global fit to the heterogeneous ligand model for M1-IgG1. Deviations from the simplest 1-1 interaction model can be observed especially if the interactions are more complex which can be a result of conformational changes after the analyte interaction with the ligand or specifically 2-state interactions. Heterogeneous ligand model is observed especially if a ligand is carrying more than one binding sites for the analyte. Therefore, we evaluated two binding sites on M1-IgG1 complex where one of them had slightly lower affinity (3,19x10^-8^M and 4,31x10^-8^ M) (S3 Table).
