# Supplementary figures and images for: Structural determination of Streptococcus pyogenes M1 protein interactions with human immunoglobulin G using integrative structural biology
Source: PLoS Comput Biol. 2021 Jan 7;17(1):e1008169. doi: 10.1371/journal.pcbi.1008169 (PMC7817036; doi:10.1371/journal.pcbi.1008169)

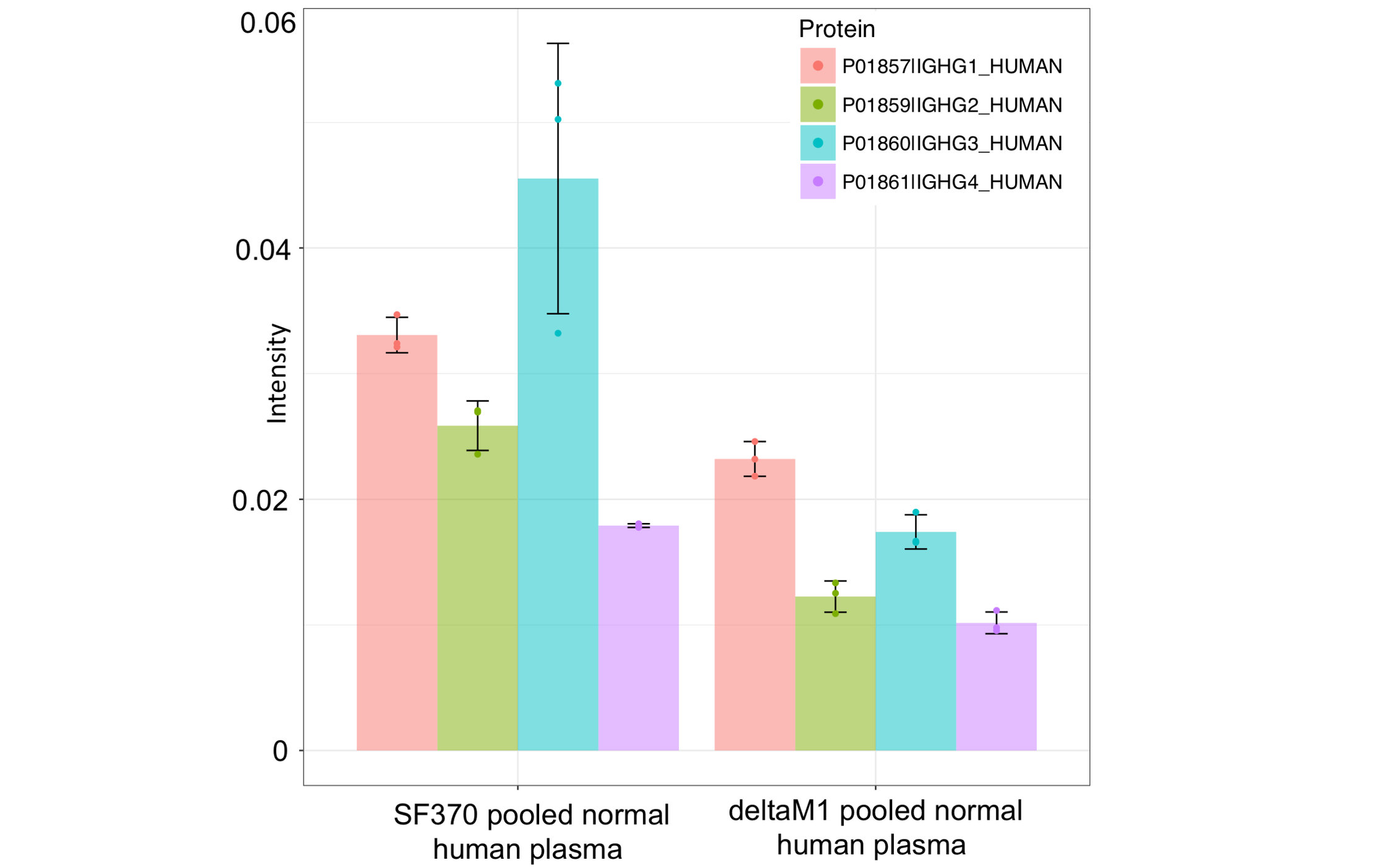

Supplement: S1 Fig — The intensity level of the heavy chains of human IgGs is analyzed through a DIA-MS analysis approach. Two groups of samples are considered here: pooled normal human plasma on the surface of the wt strain SF370 and on the surface of an SF370-derived M1 mutant strain (deltaM1). The heavy chains of IgG3 and IgG4 (IGHG3 and IGHG4, respectively) have the highest and the lowest intensities among all IgG subclasses. The data also indicates that S. pyogenes has a high affinity for IgG1 and IgG3, which is M1-mediated. (TIF) [file pcbi.1008169.s006.tif]

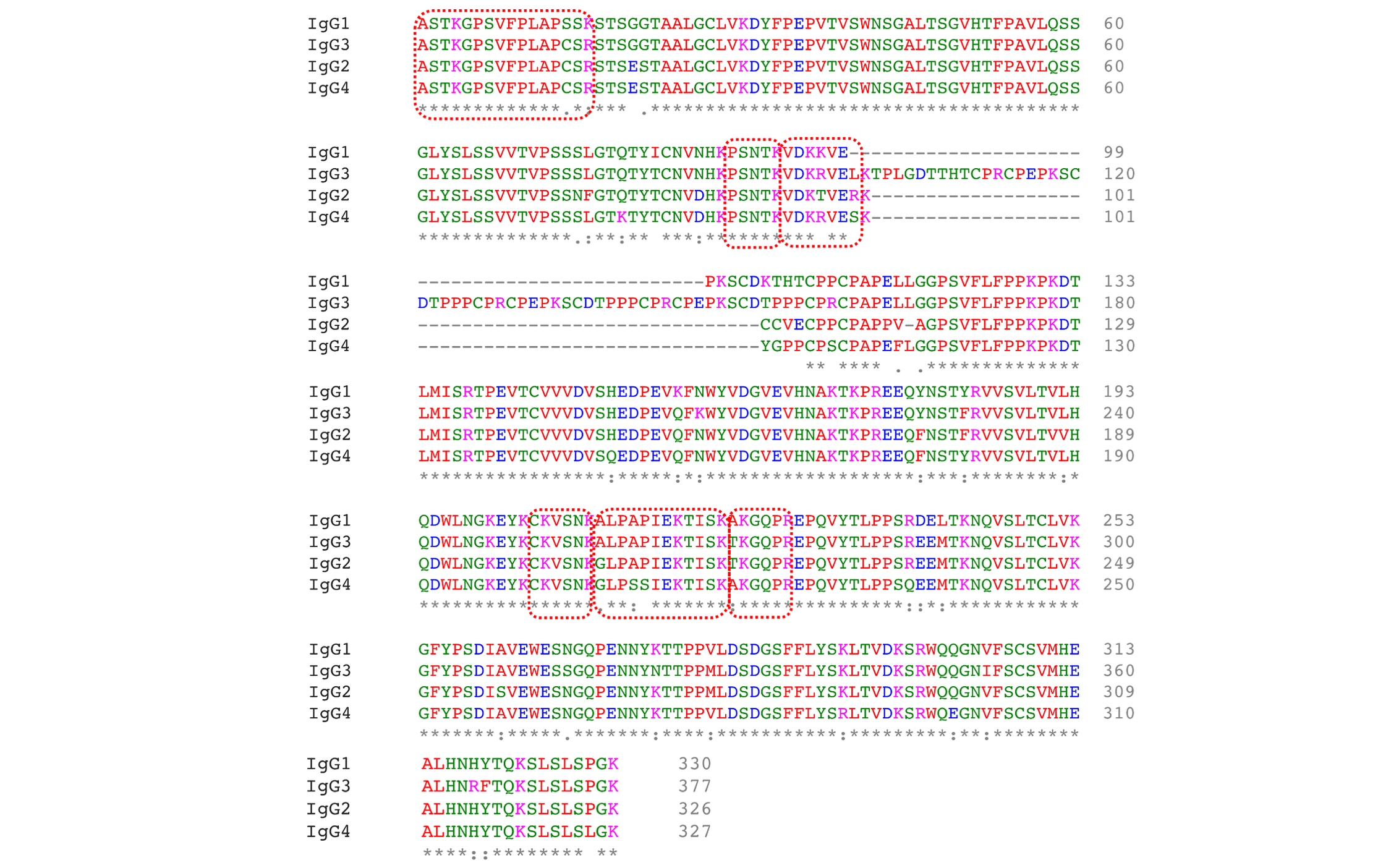

Supplement: S2 Fig — Identified XLs are shown with red dashed boxes where small sequence differences can be seen. The main difference can be noticed between IgG3 and other subclasses as the longer hinge region of IgG3 (residues 100–150 in IgG3). (TIF) [file pcbi.1008169.s007.tif]

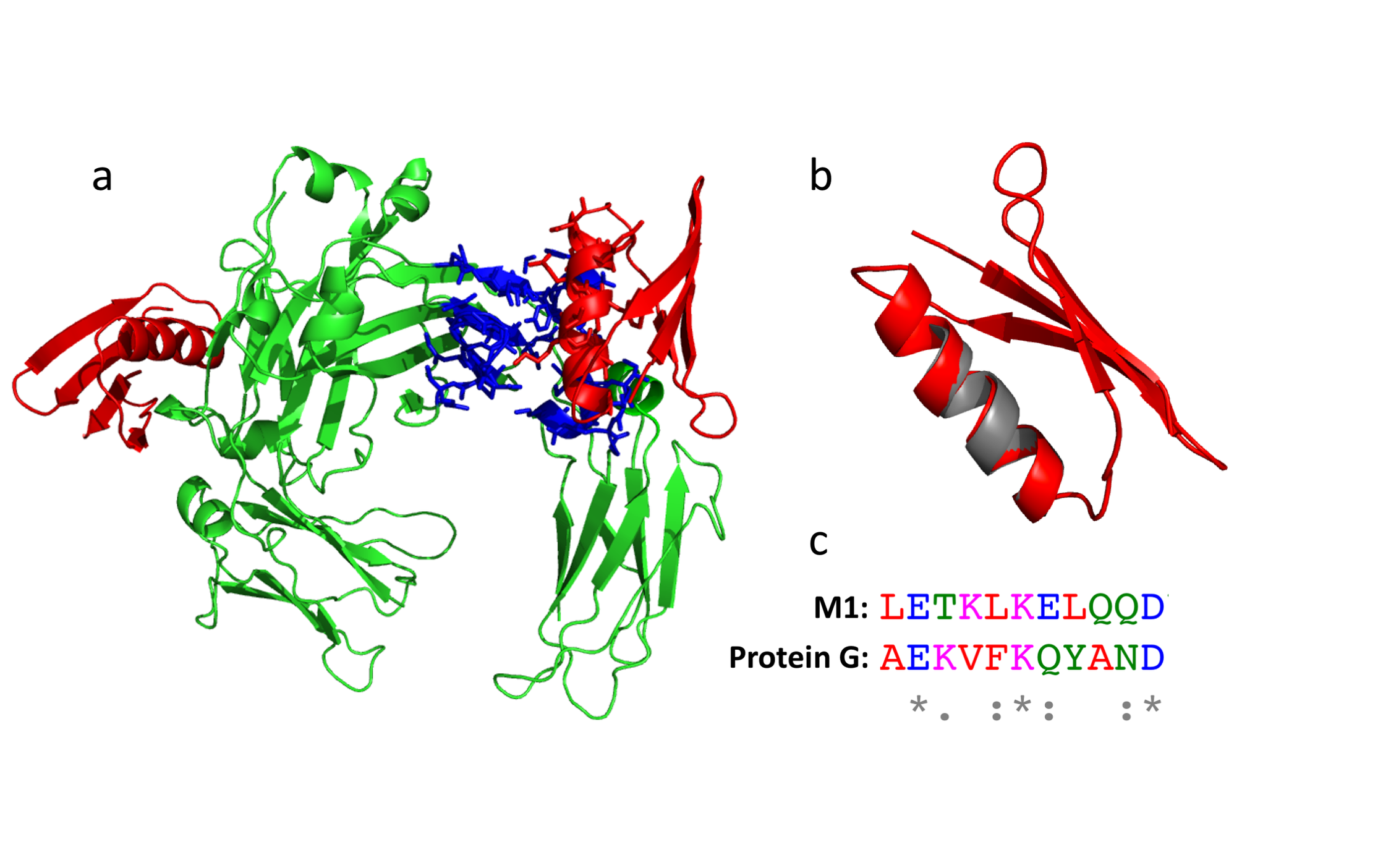

Supplement: S3 Fig — (a) Crystal structure of protein G and human IgG1 (PDB id 1fcc). (b-c) Structural and sequence alignment of protein G helix (in red) on the peptide from the M1-A domain (in grey) detected by cross-linking mass spectrometry as the high-affinity peptide to bind IgGs. (TIF) [file pcbi.1008169.s008.tif]

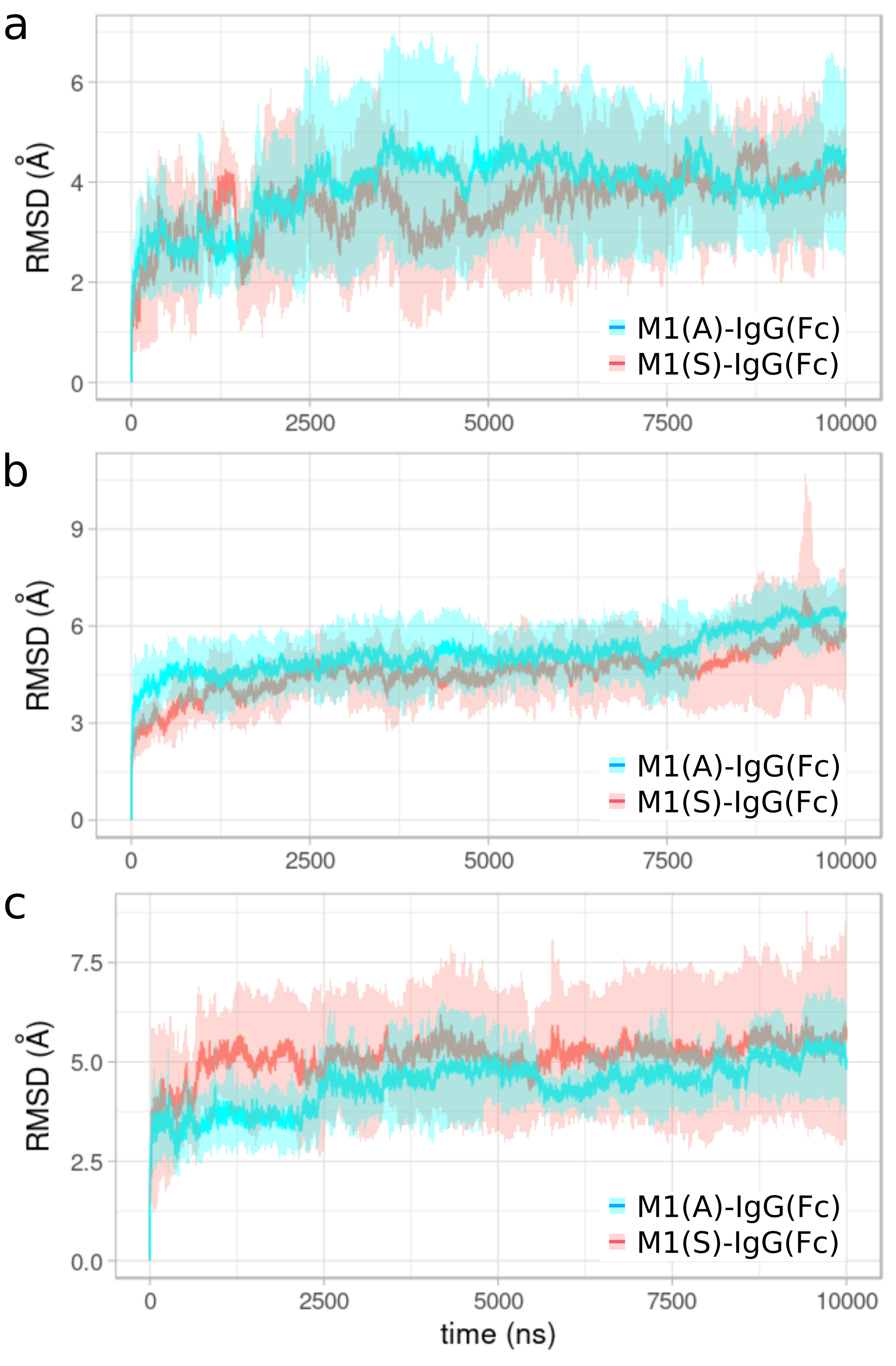

Supplement: S4 Fig — The RMSD from the equilibrated structure is computed on the backbone (C, Ca, N, O) atoms and averaged over all the five replicates of (a) the M1 peptides and (b and c) the two chains of Fc. The average values are shown as lines and the shades correspond to the standard deviations, with blue for M1(A)-IgG(Fc) and red for M1(S)-IgG(Fc). (TIF) [file pcbi.1008169.s009.tif]

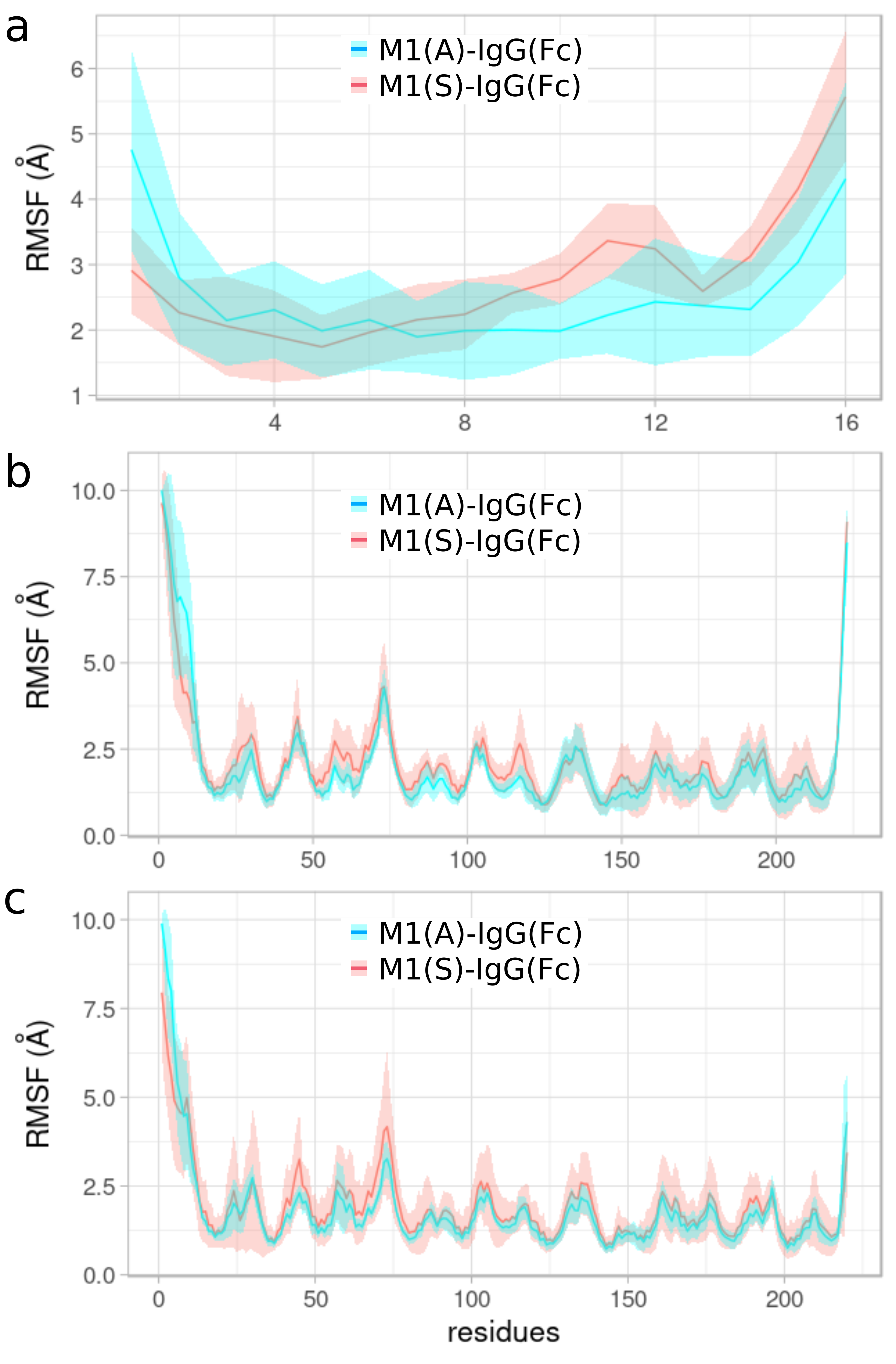

Supplement: S5 Fig — The RMSF was measured on the backbone (C, Ca, N, O) atoms with respect to the average conformation and averaged by residue, considering the last 900 ns of the MD simulations for (a) the M1 peptides and (b-c) the two symmetrical chains of Fc. The values are averaged over the five replicates of M1(A)-IgG(Fc) in blue and M1(S)-IgG(Fc) in red. The average values are shown as lines and the shades correspond to the standard deviations. (TIF) [file pcbi.1008169.s010.tif]

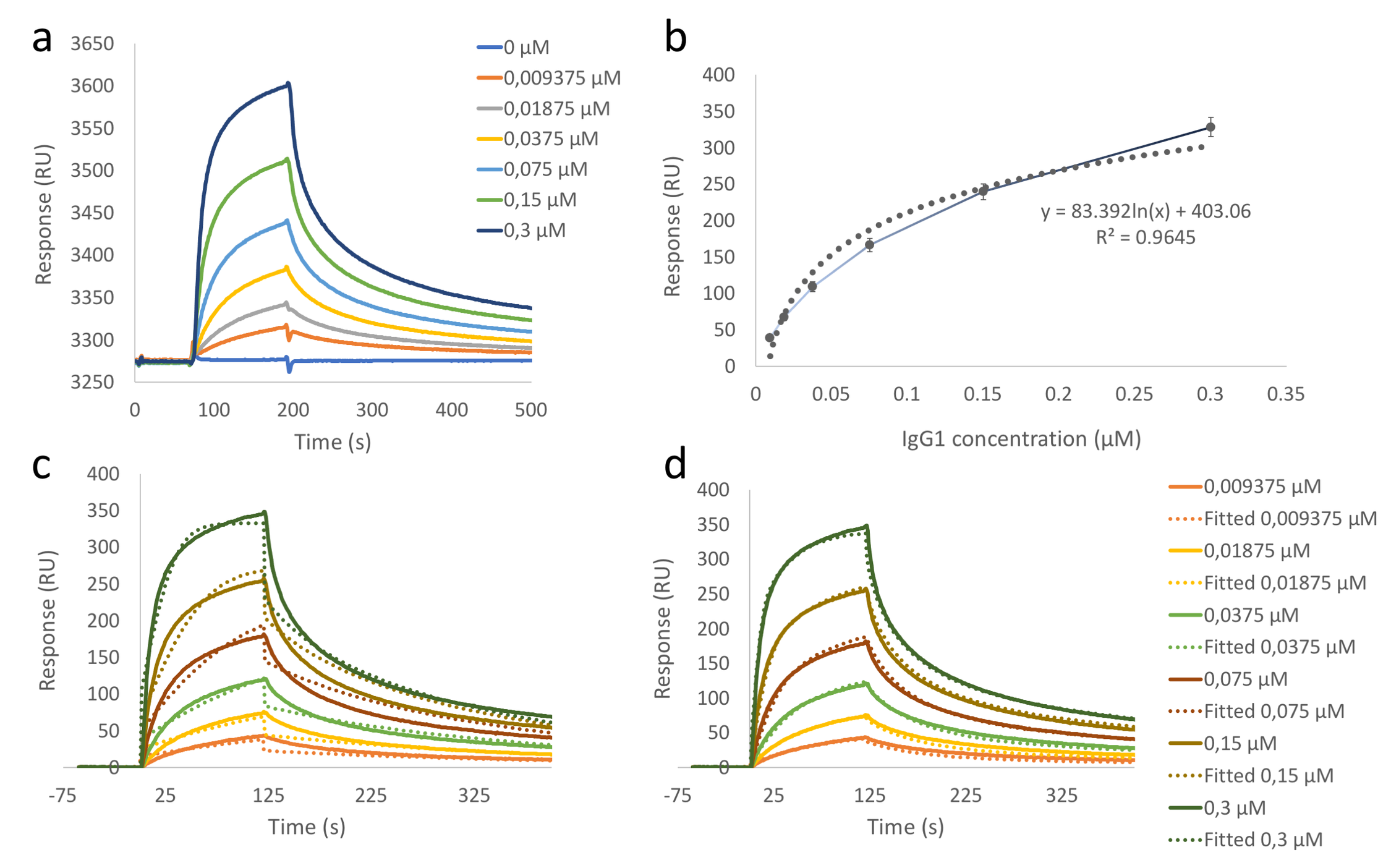

Supplement: S6 Fig — (a) Sensorgrams representing the response unit (Y-axis) plotted as a function of time (X-axis) for IgG1 binding to immobilized M1. (b) Calibration curve that shows the response unit (RU)(Y-axis) vs. IgG1 concentration (X-axis). (c-d) Kinetic analysis of IgG1 binding to immobilized M1 fitted to different models. (c) IgG1 binding fitted to 1–1 model, and (d) IgG1 binding fitted to heterogeneous ligand model. (TIF) [file pcbi.1008169.s011.tif]
